# Arachidonic acid containing phosphatidylcholine increases due to microglial activation in ipsilateral spinal dorsal horn following spared sciatic nerve injury

**DOI:** 10.1371/journal.pone.0177595

**Published:** 2017-05-24

**Authors:** Tomohiro Banno, Takao Omura, Noritaka Masaki, Hideyuki Arima, Dongmin Xu, Ayako Okamoto, Michael Costigan, Alban Latremoliere, Yukihiro Matsuyama, Mitsutoshi Setou

**Affiliations:** 1 Department of Orthopaedic Surgery, Hamamatsu University School of Medicine, Hamamatsu, Shizuoka, Japan; 2 Department of Cellular and Molecular Anatomy, Hamamatsu University School of Medicine, Hamamatsu, Shizuoka, Japan; 3 International Mass Imaging Center, Hamamatsu University School of Medicine, Hamamatsu, Shizuoka, Japan; 4 F.M. Kirby Neurobiology Center, Boston Children's Hospital and Harvard Medical School, Boston, Massachusetts, United States of America; 5 Department of Systems Molecular Anatomy, Institute for Medical Photonics Research, Preeminent Medical Photonics Education & Research Center, Hamamatsu University School of Medicine, Hamamatsu, Shizuoka, Japan; 6 Department of Anatomy, The University of Hong Kong, Pokfulam, Hong Kong SAR, China; 7 Division of Neural Systematics, National Institute for Physiological Sciences, Myodaiji, Okazaki, Aichi, Japan; University of PECS Medical School, HUNGARY

## Abstract

Peripheral nerve injury induces substantial molecular changes in the somatosensory system that leads to maladaptive plasticity and cause neuropathic pain. Understanding the molecular pathways responsible for the development of neuropathic pain is essential to the development of novel rationally designed therapeutics. Although lipids make up to half of the dry weight of the spinal cord, their relation with the development of neuropathic pain is poorly understood. We aimed to elucidate the regulation of spinal lipids in response to neuropathic peripheral nerve injury in mice by utilizing matrix-assisted laser desorption/ionization imaging mass spectrometry, which allows visualization of lipid distribution within the cord. We found that arachidonic acid (AA) containing [PC(diacyl-16:0/20:4)+K]^+^ was increased temporarily at superficial ipsilateral dorsal horn seven days after spared nerve injury (SNI). The spatiotemporal changes in lipid concentration resembled microglia activation as defined by ionized calcium binding adaptor molecule 1 (Iba1) immunohistochemistry. Suppression of microglial function through minocycline administration resulted in attenuation of hypersensitivity and reduces [PC(diacyl-16:0/20:4)+K]^+^ elevation in the spinal dorsal horn. These data suggested that AA containing [PC(diacyl-16:0/20:4)+K]^+^ is related to hypersensitivity evoked by SNI and implicate microglial cell activation in this lipid production.

## Introduction

Lipids are the most common biomolecules found in the spinal cord making approximately 50% of its dry weight [1]. These diverse molecules are involved in many cellular functions including regulation of physical properties of cellular membrane and neurotransmitter signaling [2]. Phosphatidylcholine (PC) is a major component of most intracellular membranes and is metabolized into downstream signaling lipids, such as phosphatidic acid (PA), diacylglycerol, lyso-PC, and arachidonic acid [3]. Lipid molecules such as prostaglandins (PG) [4], lysophosphatidic acid (LPA) [5], and cannabinoids [6] play an important role in the development of chronic pain, notably neuropathic pain which can occur after peripheral nerve injury in patients. Blocking PG synthesis is widely used to treat chronic, inflammatory, and postoperative pain [7]. Arachidonic acid (AA) plays a critical role in the development of neuropathic pain as the main precursor of PG [8] and of leukotriene (LT). AA has also been shown to regulate glutamate concentrations in spinal cord [9]. After peripheral nerve injury, LPA contributes to neuropathic pain by activating microglia within the spinal cord [5]. However it is not known whether PC itself in the spinal cord directly contributes to the development of neuropathic pain.

Matrix-assisted laser desorption/ionization imaging mass spectrometry (MALDI-IMS) is a reliable method to visualize a wide range of metabolites with high sensitivity and spatial resolution by determining the differences in the mass-to-charge ratios (*m/z*) of each fatty acid simultaneously [10–13]. MALDI-IMS can be used to visualize the distribution of phospholipids in injured CNS tissues such as in spinal cord [14] or ischemic brain injury [15]. Although we recently demonstrated that after nerve transection alone, arachidonic acid-containing phosphatidylcholine (PC), was significantly increased in the ipsilateral ventral and dorsal horns of the spinal cord [16], no work has been performed on the distribution of phospholipids in the injured spinal cord from preclinical models of neuropathic pain with parallel behavioral analyses.

The purpose of this work was to further analyze spatiotemporal alteration of phospholipids in the mouse dorsal horn in the SNI neuropathic pain model using MALDI-IMS and analyze how activation and suppression of the glial cell affects lipid expression.

## Materials and methods

### Chemicals

Methanol, potassium acetate, and ultrapure water were purchased from Wako Pure Chemical Industries (Osaka, Japan). Calibration standard peptides and 2,5-dihydroxybenzoic acid (DHB), a MALDI matrix, were purchased from Bruker Daltonics (Billerica, MA, USA). The antibiotic Bactramin was purchased from Chugai Pharmaceutical Co., Ltd. (Tokyo, Japan). Minocycline hydrochloride was purchased from Sigma (St. Louis, MO, USA). All chemicals used in this study were of the highest purity available.

### Glycerophospholipid naming

The side chain structures of the glycerophospholipid species are indicated within parentheses in the following format using stereospecifically numbered (sn): head group (coupling scheme-sn1/sn2); e.g., phosphatidylcholine (PC) (diacyl-16:0/18:1).

### Animals

Eight-week-old male C57BL/6JJmsSlc mice (16–21 g) purchased from SLC Inc. (Hamamatsu, Japan) were used in this study. The mice were monitored at least once per day. They were housed under controlled conditions of temperature (23±1°C), relative humidity (50%±10%), and 12-hour light/dark cycle (lights on from 7 AM to 7 PM), with free access to food and water. We had in place a protocol for early/humane endpoints as follows: weight loss greater than 20% in a day as an indicator of distress or suffering in rodents, arched position and prominence of the spine as a sign of poor body condition/sickness, excessive grooming/flinching of the animal, or vocalization, and torpor. If one animal displayed one or more of the endpoints during the daily checking, we would euthanize it immediately. All procedures were approved and performed in accordance with the guidelines for animal experimentation/care and ethics committee of the Hamamatsu University School of Medicine.

### Animal models

SNI models were performed as described in Decosterd and Woolf [17]. Briefly, mice were deeply anesthetized with ketamine 100mg/kg and xylazine 10mg/kg, then the tibial and common peroneal branches of the sciatic nerve were ligated with a silk suture and transected distally, whereas the sural nerve was left intact. In the sham controls, the sciatic nerve and its branches were exposed without any lesion. Following all procedures the animals were moved to a recovery cage and observed until they were fully ambulatory and able to take food and water, then transferred to a cage embedded on soft sawdust with free access to food and water. Post-surgery, the animals were not treated with drugs to reduce hypersensitivity as this would confound our data which aims to reveal the mechanisms of neuropathic pain. Mice were handled gently to ensure that animal distress is minimized or eliminated during examination.

The spatiotemporal changes of PCs were evaluated semi-quantitatively by MALDI-IMS in spinal dorsal horns ipsilateral to SNI or Sham injury (18 mice per group). Mice from each group were sacrificed on days 3, 7, and 21 for histological analysis (6 mice per each period).

### Minocycline treatment

A group of mice distinct from those described above received intraperitoneal (IP) injections of either vehicle (control group; n = 6) or minocycline (50 mg/kg) dissolved in sterile water (Mino group; n = 6) an hour before SNI, followed by single daily injection for seven days. These mice were sacrificed after 7 days for histological analysis.

### Behavior

After habituation and baseline sensitivity measurements, mice were examined for 3 weeks post SNI. Mice were placed on an elevated wire grid and following a 15 minute habituation period, the lateral portion of the plantar surface of the left hindpaw was stimulated using von Frey monofilaments (Touch Test; North Coast Medical Inc. Gilroy, CA). A positive response was defined as a brisk withdrawal or licking of their hind-paw upon stimulus presentation. The threshold was taken as the lowest force that evoked a positive response to one of five repetitive stimuli [18]. To ensure unbiased evaluation, all behavior tests were performed twice by a blinded examiner. For the minocycline experiment, the mice were examined until 7 days after SNI.

### Tissue preparation

Mice were sacrificed with a controlled overdose of pentobarbital sodium (25 mg/kg i.p.) and 1 cm segment of the spinal cord including L4 level was extracted. The spinal cords were embedded in CMC solution immediately after dissection and were flesh frozen in dry ice and stored at −80°C. Tissue sections (10μm) were cut at -20°C using a cryostat (CM1950; Leica, Wetzler, Germany) and placed alternately onto glass slides coated with indium-tin-oxide (ITO) (Bruker Daltonics) for MALDI-IMS, and onto un-coated glass slides (Matsunami, Osaka, Japan) for immunochemistry. For staining consistency, the spinal sections of SNI mice and sham mice were placed onto the same slides.

### MALDI-IMS analysis and PC identification

A 2,5-DHB solution (50 mg/mL DHB, 20 mM potassium acetate, 70% MetOH) was used as the matrix solution to detect signals of PC molecular species (PCs). The matrix solution (approximately 1mL) was sprayed over the tissue surface using a 0.2-mm nozzle caliber airbrush (ProCon Boy FWA Platinum; Mr. Hobby, Tokyo, Japan). Tissue sections were spray-coated with the matrix solution in order to perform extraction and co-crystallization simultaneously. The distance between the nozzle tip and tissue surface was maintained at 10 cm, and the spraying period was fixed at 20 min. Bradykinin and angiotensin-II (Sigma-Aldrich, Tokyo, Japan) were used as external calibration standards. IMS analysis was performed using a MALDI time-of-flight (TOF)/TOF-type instrument (ultraflex II TOF/TOF; Bruker Daltonics) equipped with a 355-nm Nd: YAG laser. Data were acquired in the positive reflectron mode under an accelerating potential of 25 kV using an external calibration method. Signals between *m/z* 500 and 900 were collected, and raster scans of tissue surfaces were automatically obtained at a spatial resolution of 25 μm using the flexControl and flexImaging 4.0 software (Bruker Daltonics). The number of laser irradiations was 200 shots at each location, and ion image reconstruction was performed with flexImaging 4.0 software. Signal intensity was represented about each PCs as relative intensity to the most abundant signal, [PC(diacyl-16:0/18:1)+K]^+^ in the control group.

### Immunochemistry

Sections were fixed with 2% PFA for 15 minutes, washed three times with PBS, then blocked in [0.5% bovine albumin (Sigma-Aldrich)/1% blocking reagent (Roche, Penzberg, Germany)/0.1%Triton X-100 in PBS] then incubated with anti-ionized calcium binding adapter molecule 1 (Iba1) antibody (1:500; Wako Pure Chemical Industries), and chicken anti-glial fibrillary acidic protein (GFAP) antibody (1:1000; Abcam, Cambridge, United Kingdom) overnight at 4°C. After three washes, sections were incubated with Alexa Fluor 488 donkey anti-rabbit IgG (Life Technologies, Carlsbad, CA, USA) or Alexa Fluor 594 goat anti-chicken IgG (Life Technologies) for 60 min at room temperature. Slides were then washed and cover slips were mounted onto the slides using a solution of VECTASHIELD Mounting Medium (Funakoshi, Tokyo, Japan). All images were obtained using a confocal microscope, FV1000-D (OLYMPUS, Tokyo, Japan) or a scanner, NanoZoomer 2.0HT (Hamamatsu, Shizuoka, Japan).

### Statistical analysis

All values are expressed as mean ± SEM for at least six animals per group. The behavioral data was analyzed using two-way ANOVA with Tukey. P < 0.05 was considered as significant. The statistical analyses were performed using the Statistical Package for the Social Sciences (SPSS) software (version 21.0; IBM, New York, USA).

For MALDI-IMS analysis, we converted the dataset to the ANALYZE 7.5 format file in flexImaging and analyzed using SIMtool software (in-house software; Shimadzu, Kyoto, Japan). Regions of interest (ROIs) were determined by comparison between ipsilateral and contralateral dorsal horn for both SNI vs Sham and Minocycline vs Control groups. The signal intensities in each ROI were statistically compared by Welch’s t test. Differences with p<0.05 were considered significant.

## Results

### Characteristics and distributions of primary PC species in the spinal cord following SNI and sham injury

MALDI-IMS analysis of the spinal cord tissue resembled characteristic PC distribution patterns as previously reported [14, 19]. After confirming the mechanical hypersensitivity post SNI (S1 Fig), we measured the mean signal intensities of the lesioned level of the entire spinal cord sections 3, 7, and 21 days after SNI using MALDI-IMS in the positive ion detection mode. Approximately 150 mass peaks in the range of 700<*m*/*z*<880 were detected through the entire spinal cord. Among these peaks, 11 intense peaks were assigned as PCs, taking into account their masses and previous reports [19, 20]. The peaks at *m*/*z* 770, 772, 798, 820, 824, 826, 844, 846, 848, 870, and 872 were assigned as [PC(diacyl-16:0/16:1)+K]^+^, [PC(diacyl-16:0/16:0)+K]^+^, [PC(diacyl-16:0/18:1)+K]^+^, [PC(diacyl-16:0/20:4)+K]^+^, [PC(diacyl-18:1/18:1)+K]^+^, [PC(diacyl-18:0/18:1)+K]^+^, [PC(diacyl-16:0/22:6)+K]^+^, [PC(diacyl-18:1/20:4)+K]^+^, [PC(diacyl-18:0/20:4)+K]^+^, [PC(diacyl-18:1/22:6)+K]^+^, [PC(diacyl-18:0/22:6)+K]^+^, respectively.

When the entire amount for each PC was measured and averaged for the whole spinal cord without taking into account the detailed location, the characteristic of the two spectra between the SNI and the sham group was similar (S2 Fig).

### PCs distribution of the spinal cord 7days after SNI

**Fig 1:**
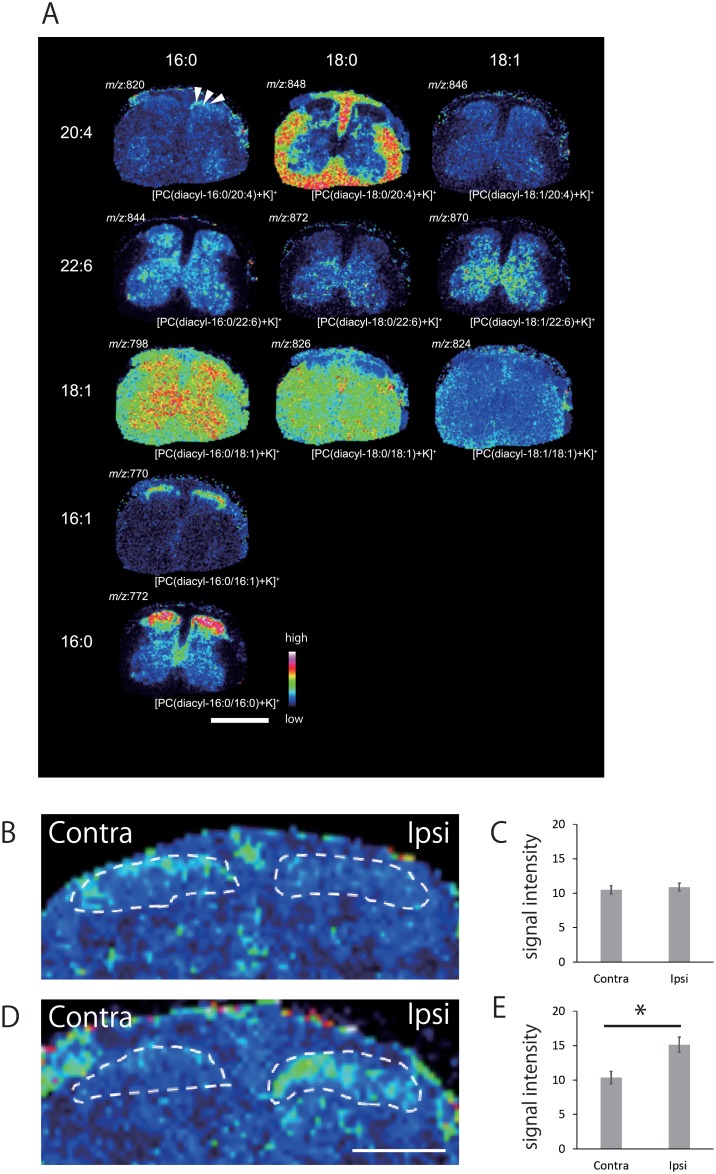
**(A) Differential distribution of PCs in the spinal cord section obtained from 7 days after SNI mice**. PCs were visualized as ion image of spinal cord section. PCs with identical FA compositions at the sn-1 position are placed horizontally, while those with identical FA compositions at the sn-2 position are arranged vertically. [PC(diacyl-16:0/20:4)+K]^+^ showed specific increase in the ipsilateral dorsal horn compared with the contralateral side (arrowheads). The other PCs showed no significant elevation in the ipsilateral side. Scale bar: 1mm. **(B~E) Statistical analysis for [PC(diacyl-16:0/20:4)+K]**^**+**^ **distribution in the spinal dorsal horn section at 7 days after the initial operation.** No changes were observed in sham group **(B, C)**. After SNI, ion signal intensities significantly increased in the ipsilateral dorsal horn compared with the contralateral side **(D, E)**. This increase was present in every animal of both groups. *p<0.05 Welch’s t test. (n = 4 for both SNI and sham). All error bars represent SEM. Scale bar: 500μm

In order to compare the spatial distributions of the 11 major PCs, in spinal cord, ion images were reconstructed using MALDI-IMS data of 7 days post-surgery (Fig 1A). The images are arranged in accordance with the two side chain structures of the PCs. Basically, PCs sharing identical FA compositions at the sn-1 position are placed horizontally, and the PCs sharing identical FA compositions at the sn-2 position are placed vertically.

### Arachidonic acid (AA) containing PC; [PC(diacyl-16:0/20:4)+K]^+^ increased at ipsilateral dorsal horn 7 days after SNI

**Fig 2:**
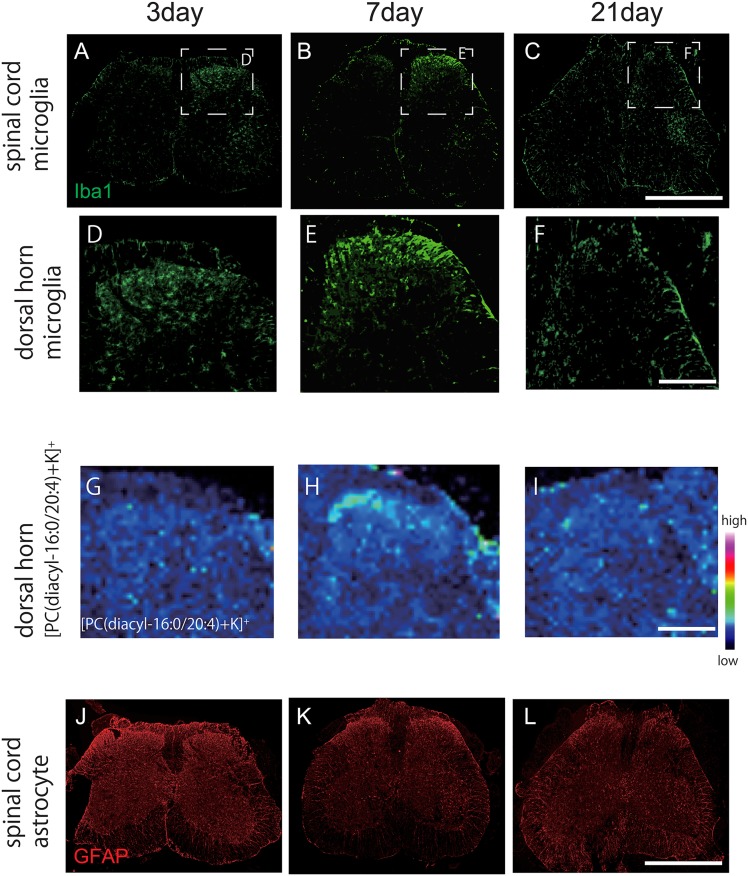
The changes of microglia and astrocyte in the spinal cord sections after SNI in comparison with [PC(diacyl-16:0/20:4)+K]^+^. The intensity of Iba-1 in the ipsilateral dorsal horn increased from day 3 (maximum at day 7) **(A~C)**, while the GFAP did not show any difference at each time period **(J~L)**. Magnified images of Iba1 **(D~F)** and [PC(diacyl-16:0/20:4)+K]^+^ **(G~I)** in the ipsilateral dorsal horn. Scale bar: 300μm. Significant increase of [PC(diacyl-16:0/20:4)+K]^+^ was observed in the ipsilateral dorsal horn at day 7, which resembled the change of microglia. Scale bar: 1mm

AA containing PC (AA-PC) is considered as a source for AA, which is a crucial mediator of synaptic transmission and intracellular signaling [21]. When the exact localization of PCs was determined, [PC(diacyl-16:0/20:4)+K]^+^ showed a marked increase in the ipsilateral superficial layer of the dorsal horn 7 days after SNI, while baseline levels were minimal (Fig 1A). Semi-quantitative analysis of signal intensities of the spinal cord at analytical points within the ROI in each group (n = 4 per group) confirmed that [PC(diacyl-16:0/20:4)+K]^+^ levels increased in the ipsilateral dorsal horn compared with contralateral dorsal horn in mice subject to SNI (Fig 1D and 1E), while no significant differences were observed in sham group (Fig 1B and 1C). The other two AA-containing PCs; [PC(diacyl-18:0/20:4)+K]^+^ and [PC(diacyl-18:1/20:4)+K]^+^ showed no significant ion signal differences throughout the period of this study in SNI or sham spinal cords. The increase of [PC(diacyl-16:0/20:4)+K]^+^ seven days after SNI was transient, as no statistical difference was found on day 3 and 21 (Fig 2G–2I).

### Spatiotemporal changes of Docosahexaenoic acid (DHA) containing PCs after SNI

Docosahexaenoic acid (DHA) containing PCs (i.e. [PC(diacyl-16:0/22:6)+K]^+^, [PC(diacyl-18:0/22:6)+K]^+^, and [PC(diacyl-18:1/22:6)+K]^+^) are implicated in synaptic function through regulating transportation of membrane proteins. These PCs decrease after spinal cord injury or degenerative neuron disease such as ALS [14] and Alzheimer’s disease [22]. However, peripheral nerve injury did not cause any change in the ipsilateral dorsal horn post SNI at each time point relative to the contralateral side (Fig 1A).

### [PC(diacyl-16:0/20:4)+K]^+^ alteration is in association with microglial accumulation

Following peripheral nerve injury, astrocytes and microglia cells become activated in the spinal cord, a process stimulated by neuroinflammation called reactive gliosis [23–25]. To analyze changes in non-neuronal support cells in the spinal cord after SNI, we labeled microglia and astrocytes using anti-Iba1 and GFAP antibodies respectively. Immunoreactivity for iba-1 increased in the ipsilateral dorsal horn from 3 days after SNI, while the intensity of GFAP signal did not change throughout the period of investigation (Fig 2). However, astrocytes displayed morphological changes (e.g. hypertrophy of processes) as observed at high power magnification 7 days after SNI, indicating astrocytosis.

Next, we compared the distribution of [PC(diacyl-16:0/20:4)+K]^+^ with these glial cell markers in the dorsal horn of SNI mice and found a very similar profile between [PC(diacyl-16:0/20:4)+K]^+^ and Iba1 staining, suggesting a potential relationship between microglial activation and [PC(diacyl-16:0/20:4)+K]^+^ elevation in the dorsal horn ipsilateral to sciatic nerve damage (Fig 2).

### Behavioral study after minocycline treatment

**Fig 3:**
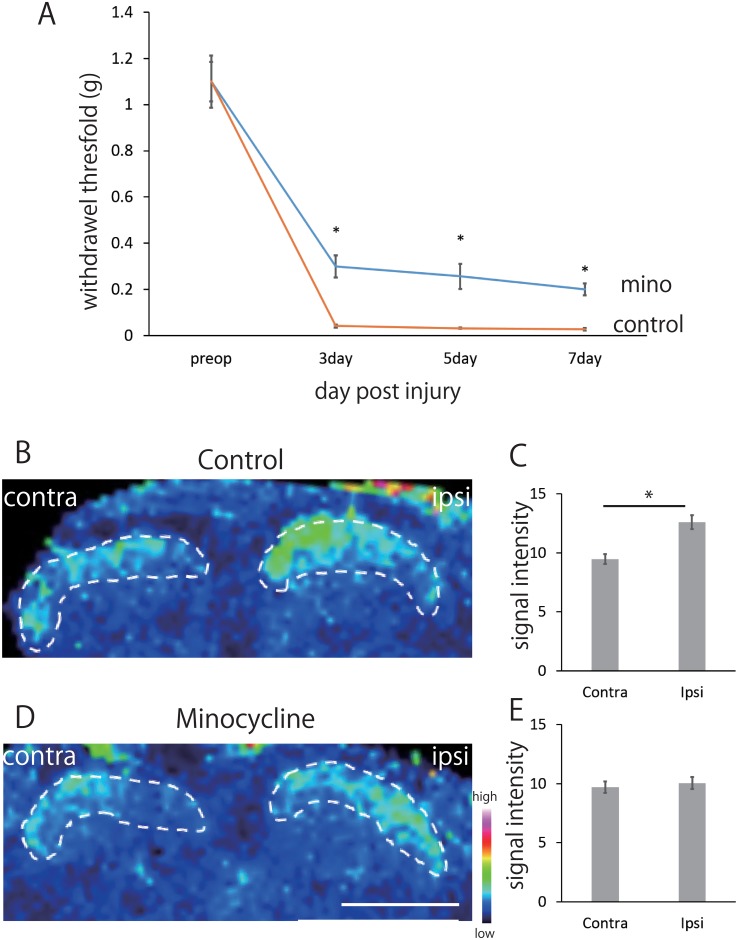
Statistical analysis for [PC(diacyl-16:0/20:4)+K]^+^ distribution with minocycline treatment. The minocycline attenuated hypersensitivity to innocuous mechanical stimulation of the left hind-paw after SNI compared with control group **(A)**. This was present in every animal and persisted until 7 days post SNI. Two-way ANOVA and Tukey. *p<0.01, minocycline versus control. All error bars represent SEM. Representative images of PC distribution in the spinal dorsal horn and quantification of the averaged ion signal intensities in each ROI in control group **(B, C)** and mino group **(D, E)**. Control group showed significant ion signal elevation in the ipsilateral dorsal horn compared with the contralateral side **(B, C)**. In mice receiving minocycline, ion signal elevation in the dorsal horn was significantly decreased **(D, E)**. This was present in every animal *p<0.05 Welch’s t test. (n = 4 for both minocycline and control). All error bars represent SEM. Scale bar: 500μm.

To test the involvement of microglial activity on [PC(diacyl-16:0/20:4)+K]^+^ elevation, we used minocycline treatment. Preemptive and daily administration of minocycline (50 mg/kg, i.p.) attenuated the hypersensitivity of ipsilateral hindpaw compared to the control mice after SNI (Fig 3A) and this persisted until at least 7 days.

### [PC(diacyl-16:0/20:4)+K]^+^ expression after minocycline treatment

Using MALDI-IMS, we investigated the mean signal intensities of [PC(diacyl-16:0/20:4)+K]^+^ 7 days after SNI in ipsilateral and contralateral dorsal horns of minocycline- and vehicle-treated mice (n = 4 per group). In control group, mean signal intensities of [PC(diacyl-16:0/20:4)+K]^+^ increased in the ipsilateral dorsal horn in comparison with the contralateral side (Fig 3B and 3C). As we hypothesized, minocycline treatment diminished the changes of [PC(diacyl-16:0/20:4)+K]^+^ induced by SNI (Fig 3D and 3E). Because minocycline selectively inhibits microglial function in the spinal cord after peripheral nerve injury [26], these results indicate that activated microglia is involved in the production of [PC(diacyl-16:0/20:4)+K]^+^.

## Discussion

Neuropathic pain has been studied though genomic [27–29], proteomic [30, 31], and lipidomic [1, 32–34] approaches. Previous studies of neuropathic pain using lipidomic approach have mainly focused on PG which is rich in fatty acids and strongly contributes to the development of neuropathic pain [4]. Recently, phospholipids such as lysophosphatidylinositol and lysophosphatidylcholine were found to be also modulators of neuropathic pain [35, 36].

Spinal dorsal horn neurons of the superficial laminae integrate and transmit nociceptive input from primary sensory fibers to the brain and they are critical for the plasticity responsible for the development of neuropathic pain, where there is a mismatch between the signal carried by sensory fibers and the information sent to the brain [37]. Various cellular and molecular mechanisms contribute to the development of a state of chronic central sensitization in dorsal horn neurons after peripheral nerve injury, and these include glutamate/NMDA receptor mediated signaling, loss of tonic inhibitory controls, and microglial activation in the dorsal horn [38]. Resident and infiltrating glia play a crucial role in modulating this pain signaling [39], therefore their status and activation within the dorsal horn is particularly relevant in chronic pain. This led us to assay lipid levels in the dorsal horn and develop an understanding which cells were expressing these changes.

In the previous study [16] we showed that after peripheral nerve injury, [PC(diacyl-16:0/20:4)+K]^+^ are increased in the ipsilateral ventral and dorsal horns in relation with reactive glia cells. However, whether the elevations of these PCs were associated with neuropathic pain behavior remained unidentified. Here, we visualize and semi-quantify PCs using MALDI-IMS in the dorsal horn and reveal the relationship with microglia in an established neuropathic model.

We successfully revealed that AA containing [PC(diacyl-16:0/20:4)+K]^+^ increased transiently 7 days post SNI and this alteration was somatotopically relevant to the injury, occurring in the superficial layer of ipsilateral dorsal horn. In our previous study using rat spinal cord injury, the elevation of AA-PCs was observed temporally 1 week following injury and also showed recruited immune cells as a possible source of AA-PC. Furthermore, MSMS analysis revealed that AA-PCs and LPC was likely to be the precursor of PG [19].

Activation of glial cells and neuro-glial interaction has emerged as a key mechanism underlying chronic pain [40]. While we observed only morphological changes in astrocytes located in the ipsilateral dorsal horn after SNI, we found a major increase in activated microglia marker iba-1 from 3 days after SNI and which persisted for at least 3 weeks, consistent with previous reports [41]. Peripheral nerve injury induces changes in microglial phenotype from a pro-inflammatory or ‘effector’ to highly mobile and phagocytic phenotype and release pro-inflammatory mediators [42]. These activated microglia upregulate COX-1 transcription, produce PG and contribute to neuropathic pain via DP2 receptor in the spinal neuron of the laminae I-II [43]. SNI induced mechanical allodynia is attenuated by COX-1 inhibition, suggesting microglial activation is one key component of neuropathic pain.

Minocycline, a semisynthetic second-generation tetracycline with adequate penetration of blood brain barrier [44], has emerged as a potent inhibitor of microglial activation and proliferation that provide release of nitric oxide (NO) metabolites and IL-1β [26]. Systemic or intrathecal administration of minocycline attenuates hyperalgesia in various neuropathic pain models, and these effects were caused by the inhibition of spinal glial activation which consequently suppressed pro-inflammatory cytokines production and release [45, 46]. Consistent with these previous studies, we show here decreased hypersensitivity in mice pre-treated with minocycline and this was associated with a decrease in the [PC(diacyl-16:0/20:4)+K]^+^ expression of the ipsilateral dorsal horn in mice receiving SNI. These results suggest that [PC(diacyl-16:0/20:4)+K]^+^ elevation in ipsilateral dorsal horn is related to the regional microglial activation and participates to the development of pain hypersensitivity after peripheral nerve injury.

PC is a major component of most intracellular membranes, and is expressed in neurons and glia. Microglia-neuron interaction plays a crucial role in the development of central sensitization by producing and releasing proinflammatory cytokines, which result in hyperexcitability of nociceptive neurons in the dorsal horn [47]. It is possible that the overproduction of [PC(diacyl-16:0/20:4)+K]^+^ by activated microglia in the dorsal horns of the spinal cord after peripheral nerve injury leads to the abnormal integration of this lipid into the membrane of spinal nociceptive neurons, which could in turn contribute to the development of pain hypersensitivity.

## Conclusions

Using MALDI-IMS, we visualized the AA containing [PC(diacyl-16:0/20:4)+K]^+^ and observed that this phospholipid was increased in the ipsilateral superficial dorsal horn 7 days after SNI. This alteration was related to the microglial activities as suppression of microglia by minocycline administration suppressed the regulation. Our results suggested that [PC(diacyl-16:0/20:4)+K]^+^ regulated by activated microglia in the spinal dorsal horn contribute to the development of hyper sensitivity after peripheral nerve injury.

## Supporting information

S1 Fig Prior to SNI, all the mice exhibited comparable baseline thresholds to mechanical stimuli (1.033g +/- 0.077g). Mice subject to SNI (n = 6) developed a marked hypersensitivity to innocuous mechanical von Frey filament stimulation of the left hindpaw after the surgery. This was present in every animal of SNI group and persisted until 21 day after surgery. No mechanical hypersensitivity was observed on the left hindpaw in the sham mice (n = 6) at any time during 3 weeks of observation. Two-way ANOVA and Tukey. *p<0.01, SNI versus sham. All error bars represent SEM.
(EPS)

S2 Fig A representative mass spectra obtained from the mouse spinal cord section 7days after surgery by MALDI-IMS. Data from the SNI mice and the sham mice 7days after operation are shown in the range of *m/z* 750 to 900. Average mass spectra in whole spine section were similar and no characteristic difference was observed between two groups.
(EPS)
